# Diseases of the Rectum and Anus

**Published:** 1837

**Authors:** 


### REVIEWS

AND

### BIBLIOGRAPHICAL NOTICES.

### Art. VI.—Diseases of the Rectum and Anus.

*A Treatise on the Malformations, Injuries and Diseases of the Rectum and Anus.* By George Bushe, M. D., formerly Professor of Anatomy and Physiology. 8vo. pp. 299—with nine quarto plates. New-York: French & Adlard. 1837.

In entering upon a review of this work, it is not without feelings of profound regret that we are compelled to announce to the reader that the able and lamented author "has gone to that bourne from whence no traveller returns." Dr. Bushe fell a victim to pulmonary phthisis early last summer, soon after the present treatise was issued from the press, in the thirty-ninth year of his age. He was a native, we believe, of Ireland, from whence he emigrated to the city of New-York, in the autumn of 1829, at the solicitation of the Medical Faculty of Rutger's College, to occupy the chair of Anatomy and Physiology, vacated some months previously by the resignation of our lamented Godman. In this situation, however, he continued only for one session, as, in the meantime, the institution with which he was connected became a complete wreck, by reason of the arbitrary and unjust interference of the State Legislature, which seems, until recently, to have always acted on the principle that one school, and one set of men, were enough for all the purposes of medical instruction in the great metropolis of the Union. How far this narrow-minded and illiberal policy, on the part of the "wise law-givers" of the State of New-York, had a tendency to promote medical education, it is not our design now to enquire. It is only necessary to state, that Dr. Bushe, having been thus early and unjustly deprived, in common with his distinguished colleagues, of his professorship, retired to private life, and soon acquired an extensive and lucrative practice, which steadily augmented until a short time prior to his demise.

Of the early history of Dr. Bushe we are entirely ignorant, nor, were we acquainted with it, would this be the place to enlarge upon it. Besides the work under consideration, he was the editor of a medical periodical, entitled the New-York Medico-Chirurgical Bulletin, and to which he contributed a large number of highly interesting essays and reviews. Like most of the medical journals, however, got up by our New-York brethren, it soon languished for want of patronage, and after the publication of the first volume, Dr. Bushe was compelled to relinquish the enterprize. In 1835, he published an "Essay on the Operation of Cleft Palate," in which he suggested some very important improvements upon the methods previously in vogue, and presented drawings of several instruments which are entirely the result of his own ingenuity. This work, designed to relieve a most unfortunate class of sufferers, has been very favorably noticed in Great Britain and on the continent of Europe, whilst, as is customary in such matters, it has been greatly neglected at home. So far, indeed, as our information extends, not a copy of it is to be found in any of the bookstores of this city; perhaps not in the whole valley of the Mississippi!

As a teacher, Dr. Bushe ranked pre-eminently high. Deeply versed in whatever related to his department, he threw around his subject an interest so fascinating as to rivet the attention of every member of his class. We well remember a lecture which he delivered on the structure of the hip-joint. Instead of confining himself to a dry detail of the descriptive anatomy of that important region, he gave a most lucid and able account of its surgical pathology; thus making one part of his discourse, as every man desirous of conveying useful instruction should always endeavor to do, subservient to the other. This lecture, the only one which we had the pleasure to hear, had but one fault—a fault not often found at the present day—that of being too learned. Dr. Bushe had a good deal of the Irish brogue; but this, if possible, only served to give additional charms to his prelections. In his person he was tall and well proportioned, with a countenance expressive of great intelligence; in his disposition, kind and affable, ever ready to minister to those in distress; and as a writer, although his style is sometimes harsh and characterized by too much haste, he had few who could excel him in vigor and rapidity. Dr. Bushe, it is presumable, had his faults; but of these we know nothing personally, and even if we did, God forbid that we should not throw over them "the mantle of charity and forgetfulness." As a lecturer, and a practitioner of surgery, he possessed qualities of no ordinary kind, and the citizens of New-York, as well as the whole American profession, have reason long to regret his premature decease.

The treatise, the title of which stands at the head of this article, is the last of Dr. Bushe's productions, but in point of interest and utility decidedly the most important to the profession, and the most creditable to his talents and erudition. As a monograph on the malformations, injuries and diseases of the rectum and anus, we know of no work in the English language which is capable of competing with it; and although some of the topics are not discussed quite so fully as could be desired, yet, regarded as a whole, it does great credit to the profession of which Dr. Bushe was so bright an ornament. We make this observation, not from any particular partiality for the memory of the distinguished author of the work before us, but from the fact that a Philadelphia reviewer has thought proper to characterize it as superficial and full of faults, chiefly, we may suppose, because it does not embrace any reference to an article—highly honorable, by the way, to his talents—which that gentleman published a year or two ago in the American Cyclopedia of Practical Medicine and Surgery. Why Dr. Bushe should have overlooked that paper, to the great annoyance of his eastern reviewer, cannot, when we consider his extensive erudition, be easily imagined; the only reason which suggests itself is the state of his health, which, as he informs us in the preface, had been gradually declining for the last twelve or eighteen months before his treatise was published, and which, no doubt, prevented him from extending his researches into works of such recent date as the one to which we have alluded. This, we conceive, should have served as a sufficient apology, and disarmed every thing like speciality in criticism. But it is high time that we should proceed with our review.

The treatise of Dr. Bushe consists of twenty-six chapters, and embraces a comprehensive account of the most important points concerning the structure, functions, malformations, injuries and diseases of the inferior portion of the intestinal canal. In presenting an analysis of the work, we shall confine our attention principally to such parts as are deemed to be of peculiar interest to the profession; or such as have been illustrated by the author's own experience and observation, referring the reader for more ample details to the book itself. As regards the structure and situation of the rectum, little need be said in this place, as all the information that may be desired on these points is to be found in our books on anatomy, especially in Cloquet, Horner, and Meckel. In speaking of the *valves* of this tube, first described by Morgagni, and again particularly noticed, about seven years ago, by Dr. Houston, of Dublin, the author positively declares that, notwithstanding the most careful investigation, he has never been able to detect them in a single instance. In our own dissections we have not been more fortunate, and are therefore inclined to agree with Dr. Bushe, that if these structures ever do exist, it is only in very rare cases, and in a very imperfect and undeveloped state. Nearly similar testimony is borne by Professor Ribes, one of the most able practical anatomists of Paris. The fear, then, of entangling bougies and other instruments in these valves, a circumstance so much talked about by some physicians, seems to be altogether groundless.

*Malformations* of the rectum and anus have been classed by the author under the following heads, to each of which he has allotted a very accurate yet concise description:—Imperforation; Unnatural termination; Absence of the Rectum. The first of these deviations from the normal state is sometimes complete, sometimes imperfect; that is to say, in the one case the closure is effected by a lamina of fibro-cellular tissue, of greater or less thickness and density; in the other, there is simply a contraction of the extremity of the rectum, or the skin is drawn over the margin of the sphincter muscle.

*Imperforation* of the rectum may take place in three different ways, first, by one partition, generally thin, transparent, and situated from two to fifteen lines above the inferior outlet; secondly, by two partitions, the structure and position of which are liable to considerable variety. An instance of this imperforation was once witnessed by Dr. Bushe in a new-born child brought into his dissecting-room. The septa were thin and pliable, about three-quarters of an inch apart, the lower one having been placed nearly six lines from the anus. The other mode in which the malformation may occur, is by puckering and induration of the walls of the rectum. This variety is extremely rare, and has never been observed by our author.

The rectum may be entirely wanting; or it may open into the bladder, urethra, vagina, or even the sacral region, from deficiency of the caudal portion of the spinal column. Occasionally the tube terminates in two extremities. Of this, Dr. Bushe once saw an interesting case in a child a few days old; one of the canals was situated a little more anterior than natural, whilst the other, though also on the middle line, was placed about an inch further back, and did not discharge more than one-third of the feces, its diameter being very contracted, and its length scarcely eighteen lines. Sometimes the vagina, or one or both ureters, open into the rectum; but this is very rare. In some cases a preternatural anus exists, occupying some situation in the abdomen, chest, neck, or even the face. Of the latter variety, a curious instance is mentioned by Bils, in his work on anatomy, published at Rotterdam, soon after the middle of the seventeenth century. Dinmore refers to a case, in which the bowel, turning upwards, opened beneath the border of the right scapula.

The majority of these malformations are, of course, fatal; the infant dying within twenty-four or seventy-two hours after birth. When the anus is closed merely by a thin covering of skin or cellulo-fibrous matter, the judicious surgeon will often be able to afford immediate relief with the knife; but the misfortune, in most cases, is, that the intervening tissue is of great extent, and the bowels consequently placed so high up as to be completely out of reach. When the rectum is partially absent, or ends in the bladder, our only resource is, after having failed in reaching it in the perinæum, to open the colon; and the portion of it that is generally selected for this purpose is the sigmoid flexure. For the steps of this operation, first performed, we believe, by the celebrated French surgeon Littre, we must refer to Dr. Bushe's work, where the reader will find all the necessary details, together with an account of the after-treatment. To this mode of getting access to the bowel, Callisen prefers cutting into the cœcum, or the descending colon in the lumbar region, along the margin of the square muscle, as by this means the peritonœum is left untouched. This operation was put to the test, some years ago, by Professor Roux, of Paris, but without succcss.

The only other operation which we shall mention in connexion with this subject is that first suggested by Dubois, and subsequently carried into effect by Martin. It consists in opening the sigmoid flexure of the large bowel, and then passing a sound through it towards the perinæum, with a view of making it prominent, and thus serving as a land-mark for our incision. This method has received the sanction of Velpeau, who recommends a sound, armed with a sharp-pointed trocar; but the operation is so cruel, that, as has been justly observed by the author, it is to be hoped that no man at the present day will attempt it.

When *foreign bodies* find their way into the rectum, or are generated within, as is not unfrequently the case, various instruments will be necessary for their removal or extraction, according to their size, shape, and situation. Amongst these, the most useful are the blunt-hook, scissors, forceps, and the canula, armed with a strong silver wire or waxed ligature. When the extraneous substance is large, very rough or spiculated, it may be proper to divide the sphincter muscle; but this measure is seldom necessary, owing to the great dilatability of the anal outlet. Should the foreign body be a piece of bone, ivory, wood or horn, fixed across the intestine, it may be extracted with a pair of lithotomy or polypus forceps, or, if it be low down, with the fore-finger. Indurated feces may be removed with a scoop or small spoon. After the pouch of the rectum is thus evacuated, the author recommends throwing up a large quantity of warm oil, for the purpose of softening down, and consequently causing the more complete expulsion of the indurated mass.

Leeches sometimes make their way into the rectum, when applied round the margin of the anus. To expel or destroy them, it is only necessary to inject a solution of salt, or an infusion of tobacco. The removal of ascarides, which so frequently infest the lower portion of the large bowel is best accomplished, according to Dr. Bushe, with a small lithotomy scoop. This method is much better than the insertion of the finger, as suggested by Mr. Howship, or the introduction of a tallow candle, as recommended by Brera.

*Laceration* of the rectum, which forms the subject of the fifth chapter, is treated with much ability, being illustrated with a number of interesting cases occurring under the author's personal observation. This accident, which is always of an unpleasant nature, may result from a great variety of causes, such, for example, as the expulsion of indurated feces, the introduction or extraction of foreign bodies, and the passage of the child during parturition. In the latter case, the rupture is often complete; in the former, generally partial, and demanding, consequently, but little surgical interference; the application of bread poultices, emollient lavements, a soluble condition of the bowels, and cleanliness, with light unirritating diet, being nearly all that is required to effect a cure.

When the laceration is complete, it is usually, if not always, produced, as was before intimated, by parturition. This accident, says Dr. Bushe, may be divided into three species, each of them presenting some peculiar features. In the first, there is merely a rupture of the sphincter muscle; in the second, the rent extends somewhat higher up; and in the third, it embraces the vagino-rectal partition, together with the sphincter and perinæum. The last of these species is much rarer than the others, and is commonly produced by the inordinate pressure of the head or buttocks of the child during parturition; these parts being directed so far backwards, that, when the uterus contracts, the recto-vaginal septum is forced downwards through the dilated anus, and then lacerated from within outwards. Our author dwells at considerable length upon the different circumstances giving rise to these lesions; but in this it is unnecessary to follow him, though many of his remarks are characterized by much novelty and ingenuity.

In the treatment of these different species of laceration, it may be stated, generally, that the patient should be confined to the recumbent position, and kept upon low diet; the lacerated parts should be constantly wet with cloths, saturated with leadwater and laudanum, and the bowels daily evacuated with emollient enemata, until suppuration is established. If there be much fever, blood may be taken from the arm; and, where the surrounding parts are much inflamed, leeches may be applied.

Sutures should never be applied immediately after the reception of such injuries, from the tendency they have to excite undue inflammation, and cut their way out through the tumefied parts. The proper period for their employment is when granulations sprout up; for Dr. Bushe affirms that in the many cases he has witnessed, he never saw one in which union by the first intention took place. This practice, we believe, to be highly judicious, and should never be lightly passed over by the surgeon. Sutures are abominable things any how, and there is much reason to think that they are often employed without any real necessity.

When the sphincter alone is lacerated, our author, instead of resorting to the interrupted suture, prefers passing a needle, of peculiar coustruction, across the bottom of the granulating wound, and then twisting a strong thread over its extremities, as in the operation for hare-lip. Should the rent be very extensive, several needles may be employed; and, additionally to these, the parts may be supported by light bodies of lint and a proper bandage. This method of operation, as Dr. Bushe informs us, was first carried into effect by Dr. Stevenson, surgeon to the New-York almshouse. When the rectum is ruptured above the sphincter, very little need be done besides the means pointed out under the head of the general treatment, that is, all that is necessary is to enforce the horizontal position, restrict the patient to a meagre diet, and preserve the bowels in a soluble state by castor oil and laxative enemata. When suppuration is established, the bowels should be quieted for a few days by the exhibition of opium; and should the wound be tardy in healing, its edges should be freely touched with the nitrate of silver.

Of the third variety of laceration our author gives an interesting case, which occurred in a married woman, in consequence of difficult labor. Thirteen months after this event, he found the surfaces of the wound separately cicatrized, and all the parts in a puckered, tumid, and excoriated state, attended with much local irritation. Having subdued this by appropriate remedial measures, he placed his patient as in the operation for lithotomy, and having pared away the edges of the sore on a wooden gorgeret, passed two sutures, half an inch apart, through the lips of the recto-vaginal septum, and then tied them in the vagina. Finally, he inserted the perinœal needle, referred to in a previous paragraph, and in the course of eight days he had the satisfaction of seeing the parts firmly re-united.

The three next chapters, treating of inflammation of the rectum, and excoriation of the anus, together with the effects arising from the application of gonorrhœal matter, are despatched in a few pages—six or seven—and present nothing of sufficient importance to detain us. Indeed, this may be regarded as the most meagre portion of the work, and it is somewhat surprising that the learned author should have slurred over these subjects in the manner he has. He surely could not have seen the erudite disquisition on the anus, in the Cyclopedia of Practical Medicine and Surgery, "published in a neighboring city, as he would there have found a much more full and perfect account."

In the ninth chapter, the author has discussed that singular disease, named by Boyer and other French writers, *fissure* of the rectum, of which he seems to have witnessed a considerable number of cases, though it is generally, and perhaps justly, considered as of very rare occurrence in this country. He describes this affection as consisting of an ulcer, from three to twelve lines in length by the eighth of an inch in breadth, situated immediately within the anus, generally on one side, sometimes in front, and ssill more rarely behind. Women, from leading a more sedentary life, are more prone to it than men; and it is seldom that persons are afflicted with it before the thirtieth or fortieth year. In the majority of cases, the fissure is confined to the mucous membrane, though occasionally it extends to the muscular substance; and its inferior extremity usually corresponds to the margin of the external sphincter, being seldom higher up or lower down. In most instances, the disease is preceded by one or more vascular tumors, and, when fully formed, is apt to be a source of much suffering and inconvenience. The pain, which is at first slight, at length becomes extremely severe, burning, lancinating, and, in severe cases, even throbbing; stimulating food or strong drink, coughing, sneezing and urinating, always increase it, and sometimes throw the poor patient into the utmost agony. Similar torments are experienced at every effort to void the feces, which, when solid, are often streaked with blood and matter. With these local affections, there is connected, of course, often more or less constitutional disturbance.

The seat of the disease can often be detected by simply separating the buttocks. Where this fails, the index-finger should be introduced, or the anus dilated with the speculum. During the treatment, the patient should be kept on light diet, be confined to his back, and have the rectum daily emptied by an enema of flax-seed tea. Stimulating cathartics, our author thinks, and we think with him, cannot be too highly censured, as being calculated to create great irritation and spasmodic action of the sphincter muscle. When these symptoms are very distressing, much advantage will be derived from an ointment, composed of one part of the extract of belladonna and seven of simple cerate, the sore being previonsly touched with lunar caustic. In mild cases, a cure may occasionally be effected with the sugar of lead salve, either alone, or in combination with belladonna, as recommended by Dupuytren. Should these measures not succeed, the only thing that remains to be done, is to divide the sphincter muscle, "which never fails to give immediate and permanent relief."

Dr. Bushe seems to have had repeated opportunities of observing *neuralgia* of the rectum, a disease first described, we believe, by Dr. Montegre, in 1812. Some of the cases which he witnessed are very curious, and we shall therefore abridge one or two for the benefit of our readers. The first we shall notice, occurred in a middle-aged physician, of active habits, in tolerable health, but of a nervous temperament, and subject to occasional attacks of neuralgia of the face, stomach and testicles. Several times a year, he would be seized with pain over the pubes, and a desire to void his urine; more rarely he suffered excruciating torments at the end of the penis, or the posterior part of the urethra, attended with a similar state of the extremity of the rectum. These attacks usually subsided in twelve or twenty-four hours, and were generally either preceded or followed by neuralgia elsewhere; No remedies were of any avail. In the other case, that of a nervous female, thirty-five years old, the pain was seated over a spot about the size of a shilling, on the left side of the bowel, rather less than half an inch above the verge of the anus. For weeks the pain would almost wholly subside, and then recur with extreme violence; her distress was generally greatest towards evening, and was always much increased during defecation. Mr. Mayo mentions* the case of a man who suffered for several years under paroxysms of neuralgia of the teeth and rectum. The attacks came on frequently during the day, without any assignable cause. In the treatment of these and other cases, Dr. Bushe tried various remedies, such as quinine, iron, arsenic, purgatives, lavements, and anodyne soppositories; but all without effect.

Passing over the eleventh chapter, on spasmodic contraction of the sphincter muscle, as containing nothing of practical value, we come to the subject of *ulceration* of the rectum, which is illustrated by six or seven cases that occurred under the author's own observation. This disease sometimes exists to a very alarming extent, the whole of the inner surface of the lower bowel being literally studded with it. When the ulcers are very small and deep, death occasionally results. Of this, Dr. Bushe once saw a case in a patient who had been treated for dysentery in India, but whose colon—the seat of this disease—was found perfectly sound on dissection. Occasionally the ulcers occur in union with stricture of the rectum, thus aggravating greatly the character of the complaint, and the sufferings of the individual.

In persons of sound constitution, the ulcers, though sometimes pretty extensive, are generally superficial, with soft, even edges. When resulting from the entanglement of feces in the lacunœ or large mucous follicles, they are usually small, somewhat circular, deep, and surrounded by a hard brim; and in unhealthy persons it is not uncommon for them to become indolent, and sometimes even phagedenic.

To detect these ulcers, the same plan of examination should be instituted as that recommended for ascertaining the presence of anal fissure. The symptoms characterizing the disease, are more or less pain in the rectum, generally of a burning, sometimes of a lancinating nature, and invariably increased by defecation; a sense of weight and fulness in the sacral region; vesical irritation, and tenesmus, with discharge of bloody and offensive matter from the anus. In bad cases the system sympathises as in fissure;

*Obs. on Injuries and Diseases of the Rectum: London, 1833, p. 56-7.

and, when the ulceration extends low down, it is not unusual to have spasm of the sphincter muscle.

Ulceration of the rectum always heals with difficulty; and for its removal requires pretty much the same treatment as fissure. When the disease is very extensive, and attended with spasm of the sphincter, much relief will accrue from the application of belladonna ointment and the nitrate of silver. Proper attention should always be paid to the constitution, which is often much impaired, and so to speak the cause of the local affection. General bleeding is rarely indicated, but where the parts are very painful, tense, and throbbing, much benefit may be expected from the employment of leeches round the margin of the anus. When all these measures fail, or afford only partial relief, the surgeon should freely divide the sphincter muscles, which often succeeds where other means prove of no avail. The following case will afford a good illustration of the author's practice in this troublesome affection. The patient, a gentleman, had discharges of bloody matter from the anus, pain in the rectum, greatly increased during defecating efforts, irritation in the bladder, and a sense of weight in the small of the back. On inspection, a circular ulcer was found, about the size of a shilling, situated above the sphincter muscle, with hard edges, and a foul, unhealthy surface. The patient was now put to bed, kept on light diet, and the bowels were evacuated daily with emollient enemata; added to which a variety of stimulating and anodyne applications were made; all, however, in vain. It now occurred to Dr. Bushe that advantage might be derived from the division of the sphincter muscles. He accordingly performed the operation, including the ulcer in the section, and then dressed the wound from the bottom. The pain instantly subsided; the parts kindly cicatrized, and in a month he had the pleasure of seeing his patient perfectly restored to health.

Of *venereal ulceration*, the next topic treated of by our author, nothing is necessary to be said in this place, as the symptoms and treatment do not differ from those in the preceding lesion, excepting that, in addition, recourse must be had to anti-syphilitic remedies. "When extensive," says he, "this species of ulceration may destroy life. In many cases the recto-vaginal partition in the female, and the recto-vesical in the male, is perforated. Provided the opening is small a spontaneous closure may ensue; but this is a very rare occurrence." Boyer refers to several cases, in which the fistula healed without surgical interference.

The most philosophical portion of the present treatise is comprised under the head of *hemorrhoidal affections*. The chapter relating to this subject is more full and extensive than any other in the book, is illustrated by numerous cases, some of them of a highly instructive character, and embraces an account of a variety of instruments and apparatus, devised or modified by the author, and designed to facilitate the performance of the different operations which are required to be executed for the removal of these tormenting maladies. Indeed, we cannot but suppose that Dr. Bushe has rendered a most important service to the profession and the public, and were all the rest of the work good for nothing—devoid of a single item of useful information—this portion would amply repay the cost of it and the time spent in its perusal.

Passing over the history of the causes and symptoms of *hemorrhoids*, with which every physician is conversant, we shall follow the author briefly in his discussion of the several varieties of the disease, and add such remarks concerning the several operations that have been recommended for its removal as may be deemed useful to the practitioner. The tumors which form about the anus, and to which writers have applied the term hemorrhoidal, may be divided into two species, one of which occupies the interior, the other the exterior of the anal outlet. The former of these are generally of a globular shape, of a dark red or livid color, according as they are free or compressed, solitary or aggragated into clusters, and in size from a small pea to a pullet's egg. In many instances, they are pedunculated, or attached by a sort of foot-stalk, especially when large, and subject to prolapsion. Their number is usually small, but now and then there are so many as to interfere, very materially, with the defecating function. "I have repeatedly," says Dr. Bushe, "injected these tumors with colored water, both from the arteries and veins, and when cut into while the fluid was projected, small jets were observed to issue from many points. I have frequently, continues he, dissected them with the greatest ease, and found that they were spongy, reddish, and contained both arteries and veins, the latter being most capacious, but always perfectly healthy. Their surface is villous, and generally bleeds when touched roughly, or scratched with the nail; the blood which issues being of a florid red color. In many instances I have been able to rub off exceedingly vascular or fragile adventitious membranes from their surfaces."

These internal tumors sometimes ulcerate; and it not unfrequently happens, moreover, that, in consequence of being inflamed and irritated, small abscesses form in them, attended with much pain, and purulent discharge. When ulceration takes place, it usually seizes on many points at once, though it rarely proceeds to any very great extent. The author once met with a case, in which three very large hemorrhoidal tumors were thus one half consumed. Occasionally, hemorrhage follows the ulcerative process; and sometimes, though this is very unusual, the tumors, although they maintain their spongy structure for years, yet it now and then happens, from their being kept in a state of constant irritation, that they are transformed into a semi-cartilaginous mass, being firm, yellow, and nearly bloodless.

"These tumors may be confounded with prolapsus of the mucous membrane of the rectum and polypi of the intestine. The form of prolapsus with which they are likely to be confounded, is that chronic affection in which a flap of the mucous membrane, on either side, is forced down and becomes thick and rugose. However, the semi-lunar form of these flaps, the extent of their base, our ability to glide the folded membrane between the finger and thumb, as well as their freedom, erection and hemorrhage, are characters so opposite to those which we have described as pertaining to hemorrhoidal tumors, that a very cursory examination enables us to distinguish between them."

In the second species, the tumors are seated round the verge of the anus, though occasionally they extend a short distance within the orifice. More or less livid, they are commonly elastic, with a broad base, and formed of extravasaled blood, which, in time, becomes *encysted* by condensed cellular tissue, and covered with fine, delicate skin from the verge of the anus, and sometimes with a few fibres of the external sphincter. In some instances, the effused blood is absorbed; but more generally it remains, and pushes out the integuments in the form of a pendulous flap, or of a rough, warty excrescence. When there is only one such tumour, especially if it be large, it not unfrequently happens that it suppurates, and shrinks.

When recent, these tumors are liable to be confounded with those of the first class, from which, however, they may be discriminated by their greater lividity, the smoothness of their surface, their hardness and freedom from hemorrhage, and, finally, by their being covered chiefly by the common skin. From partial prolapsion of the rectum, they may be distinguished not only by their density and purple color, but by their great tenderness and tubercular shape.

Such are the two classes of hemorrhoidal tumors described by Dr. Bushe, the accuracy of whose account we have had frequent opportunities of verifying both on the dead and the living. Their development seems to be often influenced by hereditary predisposition. Of this, the author has met with numerous examples, and every physician at all extensively engaged in practice must be familiar with similar cases.

"When these tumors are exceedingly painful," says Dr. Bushe, they should be anointed with the following salve, three or four times a day:

℞. Extr. Opii, gr. xii.

Ung. Cetacei ℥i.—Misce.

Sometimes the patient derives great relief from the application of cold water in a continued stream. Thus, in England, many of those afflicted with hemorrhoids are in the habit of allowing the stream which issues from the water closet, to strike against the parts prolapsed, while defecating. When the sphincter is affected spasmodically, I have found the following ointment very useful:

℞. Extr. Belladona ʒi.

Ung. Cetacei ℥i.—M.

Should the tumor be inflamed, leeches ought to be applied to the surrounding parts, followed by tepid cataplasms."

Some authors, it is well known, have recommended scarifying these tumors when inflamed; but such practice is justly condemned by Dr. Bushe, as detrimental, and likely to lead to mischievous consequences. In this we perfectly coincide with him, sure that we have seen much harm result from the measure. When the tumors protrude, and the surrounding parts are relaxed, advantage may be derived from the employment of the nut-gall ointment, so much in vogue among empirics. When there is much pain, opium or belladonna may be added; and should there be ulceration or fungous asperities, the super-acetate of lead will prove useful, in the proportion of from half a drachm to a drachm, to an ounce of water.

Should these and other means prove fruitless, the tumors must either be excised, or destroyed with the ligature; and of these two methods our author greatly prefers the latter, alleging that he has employed it with invariable success in upwards of one hundred cases. That this operation is, on the whole, more safe than the former, especially in the hands of an inexperienced practitioner, is no doubt true; but then it is so extremely painful and tedious, that we should be very loath to resort to it, except in some very rare instances. It is certainly not the most *fashionable* method with American and European surgeons, and it is one which, to us, has always appeared exceedingly awkward and bungling. The apparatus which Dr. Bushe employs for tying these tumors, consists of a pair of forceps for securing and bringing them forward, needles of different sizes, a needle-carrier, and forceps for removing the needles. All these, together with the manner of using them, are minutely described, being the invention of the author; but we have not time to give an account of them in this place.

In the treatment of external tumors, should they not be attended with much pain, the recumbent position, light diet, gentle aperients, emollient fomentations and poultices, will generally be sufficient to effect their removal. In bad cases, leeches are necessary; and if the tumor be large and elastic, the best plan will be to divide it freely with the knife, and with the scooped extremity of a director turn out the clotted blood, as there will be danger, where this is neglected, that it will suppurate. Of the advantages of this mode of procedure, several cases are related by the author.

Another species of hemorrhoids has been mentioned by writers, as consisting in a dilatation of the principal veins of the rectum. This, however, although he describes the affection, is not recognized by the author of the present treatise. The chapter in which this topic is discussed is extremely meagre, and embraces nothing whatever that is either certain or satisfactory. We shall, therefore, let it alone.

In the sixteenth chapter, we have an account of *prolapsion* of the rectum, a subject which is treated of at considerable length, and with the author's usual talent and perspicuousness. The amount of intestine dislocated, varies from a fold of the mucous membrane to several inches of the bowel itself, the protrusion being generally greatest in cases of long standing, and in children of a weakly constitution. In a case which we saw nine or ten years ago, at least nine inches of the rectum were prolapsed. In young individuals, the protruded membrane usually assumes the appearance of a small, red tumour, of a pyramidal shape; whereas in adults, it is more or less pale, and generally takes the form either of two lateral flaps, or of a circular fold. When the part is allowed to remain long prolapsed, it is liable to be engorged with blood, from the pressure of the sphincter muscles; and in old chronic cases it is not unusual for the mucous tunic to become indurated, and even ulcerated. The treatment recommended by the author, although highly judicious, embraces nothing of sufficient novelty to detain us with an account of it. Here Dr. Bushe, however, has been again guilty of the omission of a most important prophylactic means, namely, Dr. Physick's *mush and molasses plan.* This neglect, we doubt not, will be regarded by the physicians of "a neighboring city," as a most unpardonable sin, especially by the writer of the article on the anus, in the Cyclopedia of Practical Medicine and Surgery.

Of *relaxations* of the rectum and anus, the subjects treated of in the next two chapters, little more need be said than that these affections are most common in weakly children and in old and infirm persons; that they are most generally caused by habitual over-distention of the inferior portion of the large bowel, and that the best remedies are such as possess some astringent properties, as a solution of alum, sulphate of zinc, super-acetate of lead, or sulphate of copper.

Patients often suffer from what is termed *itching* of the anus, particularly such as are old or of a weakly constitution. Women who have recently ceased to menstruate, are also said to be very prone to it. The exciting causes are generally ascarides, hemorrhoidal tumors, and a morbid state of the alvine secretions. Sometimes the skin around the anus is covered with an eruption of papulæ, or even tubercles, the former of which not unfrequently vesicate, and give vent to a watery, irritating humor. Patches of a similar kind are occasionally seen on other parts of the surface, as the scrotum, thighs, back, and even the face and neck.

The pruritus, which is often very troublesome on retiring at night, so much so, indeed, as to prevent the patient, sometimes, from sleeping for hours, usually subsides after a few months, but is certain to return in consequence of the slightest irregularities in diet, fatigue, watching, or hot weather. "From constant rubbing, the skin about the anus becomes thick, dense, and furrowed, even when there are no hemorrhoidal tumors. The furrows assume a radiated direction, and converge in the anus; they vary in number from six to ten, and are from a quarter of an inch to an inch in length."

In the treatment of this affection, the first object should be to ascertain the exciting cause, and remove it. Proper attention should then be paid to the general health, which, as was previously intimated, is often much impaired. Tonics are sometimes demanded under these circumstances, such as iron, bark, or quinine, either alone, or what is still better, in combination with blue mass, sarsaparilla, and Plumber's pill; the three latter articles being particularly serviceable where there is an eruption about the anus or some parts of the cutaneous surface. In regard to the topical means which Dr. Bushe has found most useful, they comprehend the yellow-wash, a solution of lead water with laudanum, a decoction of tobacco, vinegar, tar and citron ointment, and the nitrate of silver, care being taken, when this latter substance is employed, to cleanse the parts often with water and the common turpentine soap.

The integuments around and in the neighborhood of the anus are sometimes studded with *excrescences*, which are apt to assume a considerable variety of form, and to cause a good deal of inconvenience. Generally, they are soft and fragile, smooth or figured on the surface, of a purple or pale reddish hue, imperfectly vitalized, and consequently almost devoid of sensibility. These bodies seem to be the result of inflammatory irritation of the delicate coverings of the anus; sometimes they co-exist with hemorrhoidal tumors, though usually they exist alone; and when seated within the outlet of the intestinal tube they may so close up the extremity of the gut, as to interfere with the fecal evacuation.

From hemorrhoidal tumors, these bodies may be distinguished by their color, structure, form, and incapacity of erection; from polipi, by their small size, roughness, and great number; from carcinoma, by their fragility and softness, and by the absence of hardness and ulceration of the bed from which they spring.

The surest way to destroy these tumors is to excise them with the scissors, and afterwards touching their surface with the nitrate of silver. Should the patient object to this operation, and the excrescenses are within reach, or external, then any of the following applications may be resorted to, though, there is reason to believe, that they often prove useless: lunar caustic; sulphate of copper; verdegris; oxymuriate of antimony; red oxyde of mercury; yellow wash; burnt alum, either alone, or mixed with savin powder; muriated tincture of iron; or, what the author greatly prefers to any of these substances, the strong acetic acid. When this disease depends upon a syphilitic taint, the internal exhibition of mercury and sarsaparilla will be necessary.

*Polypi* occasionally form in the rectum, more generally, it is said, in females and in adults than in the other sex or at any other period of life. In most cases, they are of a soft *mucous* substance, sessile, almost insensible, globular in their shape, from the size of a pea to that of a pullet's egg, for the most part solitary, and attached by a well-marked foot-stalk. This species of polypus is usually of slow growth, and may, therefore, be present for a long time without inducing any particular inconvenience or suffering. Not so with the *sarcomatous* species, the development of which is commonly very rapid, the bulk sometimes great, and the local and constitutional disturbance usually very considerable, the countenance being wan and sallow, the tongue coated, the body emaciated, the appetite impaired, the extremities adematous, and the system worried with hectic fever. The tenemus and weight in the rectum are often exceedingly distressing; the feces are voided with much difficulty, the pains are of a shooting, lancinating character; and there is much murco-purulent discharge, blended often with blood. If the polypus be situated near the external orifice, it will descend during defecation, and when large can only be returned with difficulty. An interesting fact in connexion with these morbid growths is, that the bowel sometimes contracts so forcibly as to detach them. The proper method of treatment consists in extraction, for which the necessary instruments are a pair of scissors, a scalpel, and Museux's forceps: the hemorrhage seldom proves troublesome.

We now come to the subject of *abscess* near the rectum; a disease which seems to have its seat originally in the cellular tissue of the part, and which almost uniformly leads to the formation of fistulæ. It may depend upon some lesion of the rectum, or it may be symptomatic of disease of the viscera, especially the liver, spleen, heart and lungs; it is very common in phthisical subjects, which may probably be accounted for by the tendency which exists in this malady to ulceration of the bowels. As the symptoms and treatment of anal abscess do not differ essentially from those of the same affection when occurring in other regions of the body, we shall here dismiss the subject, and proceed to take up the chapter which treats of anal fistulæ.

*Fistulæ*, as just stated, are usually the consequences of anal abscesses; and, in regard to their mode of communication, may be arranged into three classes; in the first, they extend into the gut; in the second, they open into the rectum, but have no outward aperture; and in the third, the fistula perforates the skin, but does not reach into the bowel. The number of external openings varies. Generally, as stated by the author, there is but one; but sometimes two, three, or even more. Internally it is rare to find more than one orifice; Dr. Ribes, however, who has carefully investigated this subject, mentions that there are sometimes two, or even three, particularly in phthisical people.

The internal orifice is generally soft and irregular; but in consumptive patients, occasionally round and callous. Its distance from the anal outlet is variable; generally, however, it is situated immediately above the spot where the internal membrane of the rectum joins the skin, sometimes a little higher, but *never* more than five or six lines. In eighty subjects examined by Professor Ribes, of Paris, the internal orifice did not exceed this height, and in a considerable number its elevation was not more than a third or fourth of an inch. These observations are confirmed, though not on so large a scale, by our author.

The external orifice varies much in its appearance, being sometimes round, sometimes oval, but more generally irregular or angular, with reddish edges, and a surface foul and depressed, or studded with exuberant granulations. The parts around the fistulæ are unusually hard, and sometimes even greatly disorganized. Frequently, especially when the disease is the result of gangrenous inflammation, a large cavity intervenes between the two apertures, which, like every fistulous track of this sort, in time becomes lined by a coat of coagulating lymph, possessing all the characters of a mucous membrane, save that it is destitute of villosities. A channel of this kind may be straight or crooked; and when there are several at the same time, they not unfrequently communicate with each other. Pus, of a thin mucous nature, generally issues from it, and, when it opens into the bowel, stercoraceous matter.

The operations for the removal of this loathsome disease are so well known, that we deem it unnecessary to describe them on the present occasion, more especially as the anthor has not added any thing new to what was previously known on the subject. He does not subscribe, as a general rule, to the opinion of Ribes and others, who assert, that, unless there is an opening into the bowel, it is never necessary to divide it, having found from experience that this method of procedure must sometimes be resorted to. When there is no such opening, compression made with a piece of cork, supported by an elastic bandage, assisted by an easy state of the bowels, and the injection of a solution of the nitrate of silver, Port wine and water, or some of the mineral astringents, together with the horizontal position, and meagre diet, will generally be sufficient for the cure. When these means fail, the knife must be employed, and the whole track laid open, and healed from the bottom.

*Stricture* of the rectum may be either functional or organic, depending, in the one case, upon spasmodic action of the muscular fibres; in the other, upon structural lesion of one or more of the tunics of the bowel. Organic stricture is a rare disease in this country, at least it is rare that we see a case in practice, or in the dissecting room. Sometimes the thickening and induration are limited to the mucous and sub-mucous textures; at others, it extends to the muscular fibres, or involves them alone. A smooth gristly ring is thus formed, from a few lines to half an inch in depth, above and below which the bowel usually retains its normal structure. The disease does not, however, always embrace the entire circumference of the gut; not unfrequently, indeed, it forms a mere segment of a circle, and is so arranged as to give the villous tunic in the immediate neighborhood a remarkably puckered and drawn-up appearance. When the ring is complete, it strongly resembles a scirrhous pylorus.

The common seat of organic stricture is about three inches above the anal orifice. Cases, however, are frequently observed, in which it is much lower down or higher up. The disease spares neither age nor sex. Dr. Bushe has observed it in a lad of nine, and in an old man of seventy. Its occasional cause is inflammation, in whatever manner induced.

The nature of this lesion can only be satisfactorily determined by an examination with the finger, and by a careful investigation of its history. The same difficulty is experienced in relieving the bowel, as in fissure and carcinoma; but what perhaps serves, in some degree, at least, to distinguish this affection from any other, is the peculiar shape of the feces, which, instead of being voided in thick masses, come away in short, narrow pieces, often not larger than a full-sized catheter. The bladder is sometimes excessively irritable, from the pressure of the loaded gut; and occasionally one or more fistulæ form in the nates or perinœum. In the female, a communication frequently takes place between the rectum and vagina; and in the male a similar passage may be established, though more rarely, between the rectum and the bladder.

This disease, if allowed progress, always proves fatal, though sometimes a long period elapses until the occurrence of this event. In the early stage of its existence, before there is any cartilaginous degeneration of the intestinal tunics, a cure may sometimes, indeed often, be effected, by a proper use of the bougie, regulating the diet, and precaution to keep the bowels, especially the rectum, constantly unloaded. The instrument recommended by Dr. Bushe, in the treatment of this affection, is a bougie, constructed of ebony, three inches long, and mounted on whale-bone, which, he says, is decidedly better than any other contrivance.

The last chapter of the present work treats of *carcinomatous degeneration* of the rectum; it is compiled solely from the materials obtained from the author's own observation, and is therefore meagre and imperfect. Appended to this chapter is a short extract from Cayol's "Traite des Maladies Cancareuses," published in Paris, in 1833, on what he styles *lymphatic engorgement* of the textures in the immediate vicinity of the anus, and which the French writer says may sometimes be confounded with carcinomatous affections. Dr. Bushe, however, never witnessed this disease, nor do we recollect any cases of it that have come under our own notice or under that of our medical friends.

We now bring our analysis to a close, and in rising from our labors we can conscientiously declare that we have been both interested and instructed. The work of Dr. Bushe is, as we have already stated, a good one, and we hope that it will soon find its way into the library of every American physician. The plates, though not splendid, are sufficiently graphic to convey an accurate idea of what they are designed to illustrate. What more, indeed, could be desired or expected of them? S. D. G.

